# PEMT Mediates Hepatitis C Virus-Induced Steatosis, Explains Genotype-Specific Phenotypes and Supports Virus Replication

**DOI:** 10.3390/ijms24108781

**Published:** 2023-05-15

**Authors:** Mosleh Abomughaid, Enoch S. E. Tay, Russell Pickford, Chandra Malladi, Scott A. Read, Jens R. Coorssen, Brian S. Gloss, Jacob George, Mark W. Douglas

**Affiliations:** 1 Storr Liver Centre, The Westmead Institute for Medical Research, The University of Sydney and Westmead Hospital, Sydney, NSW 2145, Australia; 2 Bioanalytical Mass Spectrometry Facility, Mark Wainright Analytical Centre, University of New South Wales, Sydney, NSW 2052, Australia; 3 Department of Molecular Physiology, School of Medicine, Western Sydney University, Sydney, NSW 2751, Australia; 4 Blacktown Clinical School, Western Sydney University and Blacktown Hospital, Sydney, NSW 2751, Australia; 5 Department of Biological Sciences, Faculty of Mathematics and Science, Brock University, St. Catharines, ON L2S 3A1, Canada; 6 Westmead Research Hub, Westmead Institute for Medical Research, Sydney, NSW 2145, Australia; 7 Centre for Infectious Diseases and Microbiology, Sydney Infectious Diseases Institute, The University of Sydney at Westmead Hospital, Sydney, NSW 2145, Australia

**Keywords:** hepatitis C virus, lipidomics, PEMT, phosphatidyl choline, lipid metabolism, steatosis

## Abstract

The hepatitis C virus (HCV) relies on cellular lipid pathways for virus replication and also induces liver steatosis, but the mechanisms involved are not clear. We performed a quantitative lipidomics analysis of virus-infected cells by combining high-performance thin-layer chromatography (HPTLC) and mass spectrometry, using an established HCV cell culture model and subcellular fractionation. Neutral lipid and phospholipids were increased in the HCV-infected cells; in the endoplasmic reticulum there was an ~four-fold increase in free cholesterol and an ~three-fold increase in phosphatidyl choline (*p* < 0.05). The increase in phosphatidyl choline was due to the induction of a non-canonical synthesis pathway involving phosphatidyl ethanolamine transferase (PEMT). An HCV infection induced expression of PEMT while knocking down PEMT with siRNA inhibited virus replication. As well as supporting virus replication, PEMT mediates steatosis. Consistently, HCV induced the expression of the pro-lipogenic genes SREBP 1c and DGAT1 while inhibiting the expression of MTP, promoting lipid accumulation. Knocking down PEMT reversed these changes and reduced the lipid content in virus-infected cells. Interestingly, PEMT expression was over 50% higher in liver biopsies from people infected with the HCV genotype 3 than 1, and three times higher than in people with chronic hepatitis B, suggesting that this may account for genotype-dependent differences in the prevalence of hepatic steatosis. PEMT is a key enzyme for promoting the accumulation of lipids in HCV-infected cells and supports virus replication. The induction of PEMT may account for virus genotype specific differences in hepatic steatosis.

## 1. Introduction

The hepatitis C virus (HCV) is intimately linked with the host’s lipid metabolism, which is necessary for virus replication [1]. The infectious virus is present in serum as “lipoviral particles” similar in composition to very low-density lipoprotein (VLDL), and entry of the virus into the hepatocytes is dependent on lipoprotein receptors SR-B1 and LDL-R [2,3]. Virus replication takes place in modified membranous structures known as the membranous web and relies on sphingomyelin, fatty acid and cholesterol metabolism [4,5,6,7,8]. Virus assembly occurs in proximity to cytoplasmic lipid droplets while virus secretion involves the endoplasmic reticulum (ER)–Golgi pathway similar to VLDL [9].

One approach for understanding the interactions between HCV and cell lipid metabolism is lipidomics, which characterises the changes in lipid species within cells. Shotgun lipidomics provides a detailed analysis of the composition of individual lipid species but is only semi-quantitative, limiting its reliability for inter-sample comparisons [10]. An alternative approach that we chose for this study is high-performance thin-layer chromatography (HPTLC), which provides more a precise quantitation [11].

Using HPTLC-based lipidomics, we observed a marked increase in cholesterol and phosphatidyl choline (PC) in the ER of HCV-infected cells, implicating a role for these lipid species in the virus replication cycle. We went on to identify phosphatidylethanolamine N-methyltransferase (PEMT) as the enzyme responsible for the increase in PC in HCV-infected cells through a non-canonical PC-synthesis pathway. In functional studies we showed that the HCV-induced expression of PEMT supports virus replication but also causes steatosis by modulating the expression of lipid metabolism genes. Finally, we found that PEMT could mediate the HCV genotype-specific induction of hepatic steatosis, as PEMT expression was higher in the livers of patients with genotype 3 infection than genotype 1. Our findings provide novel insights into the pathogenesis of chronic hepatitis C and its complex interactions with liver lipid metabolism.

## 2. Results

### 2.1. HCV-Induced Changes in Total Cellular Lipids

To obtain an overview of the individual lipid species and changes in their relative levels in response to HCV infection, we used high-performance thin-layer chromatography (HPTLC) to compare uninfected and HCV-infected Huh7 cells using the JFH1 cell culture model [12]. We modelled persistent HCV infection by culturing cells for at least two weeks after RNA transfection until >90% of cells expressed the viral protein NS5A. Lipid levels were normalised against protein concentration for each sample and at least three separate biological replicates were performed. Commercial preparations of the key lipid species were run in parallel as standards.

**Figure 1:**
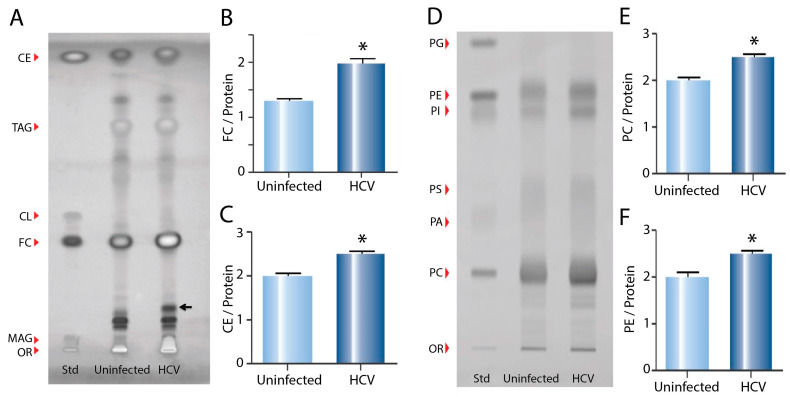
Effect of HCV infection on neutral lipid and phospholipid species in whole cell extracts. (**A**) HPTLC plate loaded with lipid standards and neutral lipids extracted from uninfected control (Huh7) and (JFH1) HCV-infected cells (OR—origin; MAG—monoacylglyceride; FC—free cholesterol; CL—cardiolipin; TAG—triacylglycerol; CE—cholesterol ester). (**B**) Quantification of FC and (**C**) CE. (**D**) HPTLC plate loaded with phospholipid standards and phospholipids extracted from uninfected control and HCV-infected cells (OR—origin; PC—phosphatidyl choline; PA—phosphatidic acid; PS—phosphatidyl serine; PI—phosphatidyl inositol; PE—phosphatidyl ethanolamine; PG—phosphatidyl glycerol). (**E**) Quantification of PC and (**F**) PE. Analysis was performed using triplicate samples from three biological replicates and normalised for protein concentration (* *p* < 0.05).

The HPTLC analysis of the neutral lipid species from whole cell extracts (Figure 1A) demonstrated increases in free cholesterol (FC) (Figure 1B) and cholesteryl ester (CE) (Figure 1C) in the infected compared to the uninfected cells, as well as an unidentified lipid species (black arrow, Figure 1A). This unidentified band was analysed using mass spectrometry, with a preliminary analysis suggesting it was a diacylglyceride.

Next, HPTLC was used to compare the phospholipid profiles of HCV-infected and uninfected Huh7 cells (Figure 1D). Quantification of the phospholipid species using Lipid Search software demonstrated significant increases in the levels of phosphatidyl choline (PC) (Figure 1E) and phosphatidyl ethanolamine (PE) (Figure 1F) in the infected cells, with no significant differences in phosphatidyl serine (PS) or phosphatidyl inositol (PI). Neither phosphatidyl glycerol (PG) nor phosphatidic acid (PA) were detected in significant amounts.

### 2.2. HCV-Induced Lipid Changes in Endoplasmic Reticulum

The endoplasmic reticulum (ER) plays a central role in the HCV replication cycle, as the membranous web containing the viral replication complex is derived from the ER [13] and viral egress occurs via the ER–Golgi pathway [14]. The ER also plays an important role in cellular lipid metabolism as the site of triglyceride synthesis and cholesterol metabolism [15]. Therefore, we analysed changes in the lipid content of the ER in response to infection. Cell lysates from HCV-infected and uninfected cells were fractionated to isolate the ER as described. The purity of ER fractions was confirmed by western blot for the ER marker protein eIF2-α (Figure S1). The lipid was extracted from the isolated ER fractions using a chloroform-methanol method and then separated and quantified using HPTLC. To allow a valid comparison among the samples, protein concentrations were measured post-lipid extraction and used to normalise the amount of lipid loaded onto each HPTLC lane. Lipids were extracted and analysed using HPTLC, then normalised against the protein concentration of the ER pellet.

**Figure 2:**
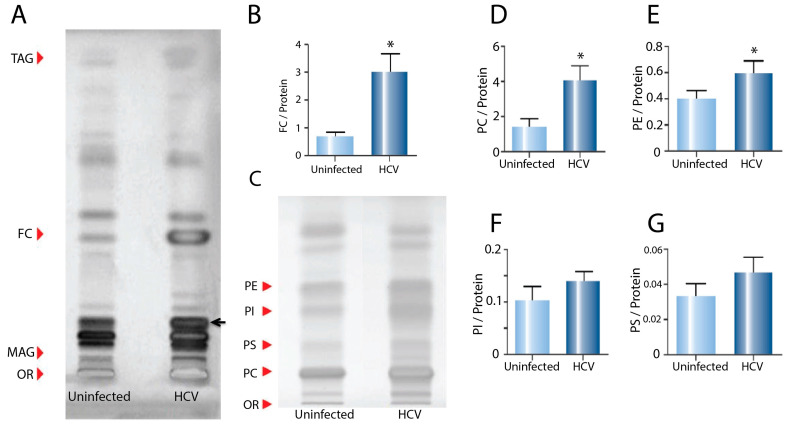
Effect of HCV infection on neutral lipid and phospholipid species in endoplasmic reticulum fractions. (**A**) HPTLC plate loaded with neutral lipids from ER extracted from uninfected control and HCV-infected cells (OR—origin; MAG—monoacylglycerol; FC—free cholesterol; TAG—triacylglycerol). (**B**) Quantification of FC in the ER of uninfected control and HCV-infected cells. (**C**) HPTLC plate loaded with phospholipid species from ER extracted from uninfected control and HCV-infected cells (OR—origin; PC—phosphatidyl choline; PS—phosphatidyl serine; PI—phosphatidyl inositol; PE—phosphatidyl ethanolamine). (**D**) Quantification of PC, (**E**) PE, (**F**) PI and (**G**) PS. Analysis was performed using triplicate samples from three biological replicates, normalised for protein concentration (* *p* < 0.05).

An analysis of the neutral lipids demonstrated a large increase in free cholesterol in the ER of the HCV-infected cells, compared to the uninfected controls (Figure 2A,B). The relative increase in free cholesterol in the ER (~four-fold) was greater than the increase in the whole cell extracts (~40%) (Figure 1B), suggesting that the increase observed in the extracts from whole cells was due to cholesterol accumulation in the ER. In addition, we observed a high intensity band in the ER from the HCV-infected cells (black arrow, Figure 2A) running at a similar position to the increased band observed in the whole cell extracts (Figure 1A). As for the whole cell extracts, mass spectrometry suggested this band was a diacylglyceride species.

Consistent with the whole cell extracts, a phospholipid profiling of the ER (Figure 2C) demonstrated significant increases in PC (Figure 2D) and PE (Figure 2E) in the infected cells. As we observed for free cholesterol, these increases were relatively greater in the ER than in the whole cell lysates (Figure 1E,F). In addition, consistent with the whole cell lysates, there was no significant difference in PI (Figure 2F) or PS (Figure 2G) in the ER from the HCV-infected cells compared to the controls.

### 2.3. Phosphatidyl Ethanolamine Transferase (PEMT) Is a Novel Host Factor Required for HCV Replication

**Figure 3:**
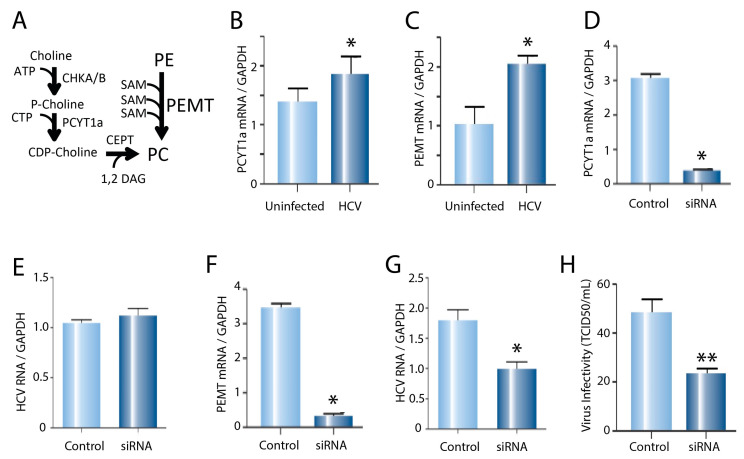
(**A**) Pathways responsible for the synthesis of PC involving PCYT1a and PEMT. (**B**) Expression in the uninfected control and HCV-infected cells of PCYT1a and (**C**) PEMT mRNA relative to the GapDH control, measured using real-time PCR (* *p* < 0.05). (**D**) Knock-down of PCYT1a or (**F**) PEMT mRNA using siRNA. (**E**) Intracellular HCV mRNA concentration 72 h after infection with the tissue-culture-derived virus, following an initial treatment for 48 h with 20 nM siRNA targeting PYCT1a or (**G**) PEMT, compared to the scrambled siRNA controls (* *p* < 0.05). (**H**) Number of infectious foci for the Huh7 cells treated with 20 nM/mL PEMT siRNA or scrambled siRNA for 48 h, then infected with the tissue-culture-derived HCV (TCID50/mL) (** *p* < 0.01).

Phosphatidyl choline is synthesised through two separate pathways: (1) from choline, via the choline dependant pathway (CDP), the rate limiting enzyme of which is phosphocholine cytidyltransferase A (PCYT1a); and (2) from PE, via an alternate pathway in the hepatocytes involving the methylation of PE by the enzyme phosphatidyl ethanolamine transferase (PEMT) [16] (Figure 3A).

To determine which pathway is responsible for the increased synthesis of PC in HCV- infected cells, expression of the genes encoding these two key enzymes (PCYT1a and PEMT) was analysed using real-time PCR. In the infected cells, the expression of both PCYT1a (Figure 3B) and PEMT (Figure 3C) was increased compared to the uninfected controls.

To assess the relative importance of each PC synthesis pathway for HCV replication, we used specific siRNAs to achieve efficient knock-down (~90%) for PCYT1a (Figure 3D) or PEMT (Figure 3F). The Huh7 cells were treated with siRNA for 48 h, then infected with cell-culture-derived HCV (JFH1 strain) at an MOI of 5. After 24 h, the virus was removed and fresh media was added. Then, 48 h later (i.e., 72 h after exposure to the virus), cells were harvested and the HCV RNA was analysed using real-time PCR to look for any effects on HCV replication. The knock-down of PCYT1a (Figure 3D) had no effect on the HCV RNA (Figure 3E), whereas the knock-down of PEMT (Figure 3F) caused an ~50% decrease in the HCV RNA (Figure 3G), suggesting that PEMT is important for virus replication. To test whether reducing PCYT1a or PEMT inhibited new HCV infection, PCYT1a or PEMT were knocked down using siRNA, then cells were infected with a concentrated JFH1 virus. After 48 h, infected cells were labelled for the virus protein using antibody against NS5A and viral infectivity was determined by the tissue culture infective dose (TCID50) assay (Figure 3H). There was an ~50% reduction in infectivity for the cells treated with PEMT siRNA (Figure 3H, *p* < 0.01), but not with PCYT1a siRNA. This suggests that PEMT is important for establishing an HCV infection and ongoing RNA replication.

### 2.4. PEMT Mediates HCV-Induced Lipid Accumulation

**Figure 4:**
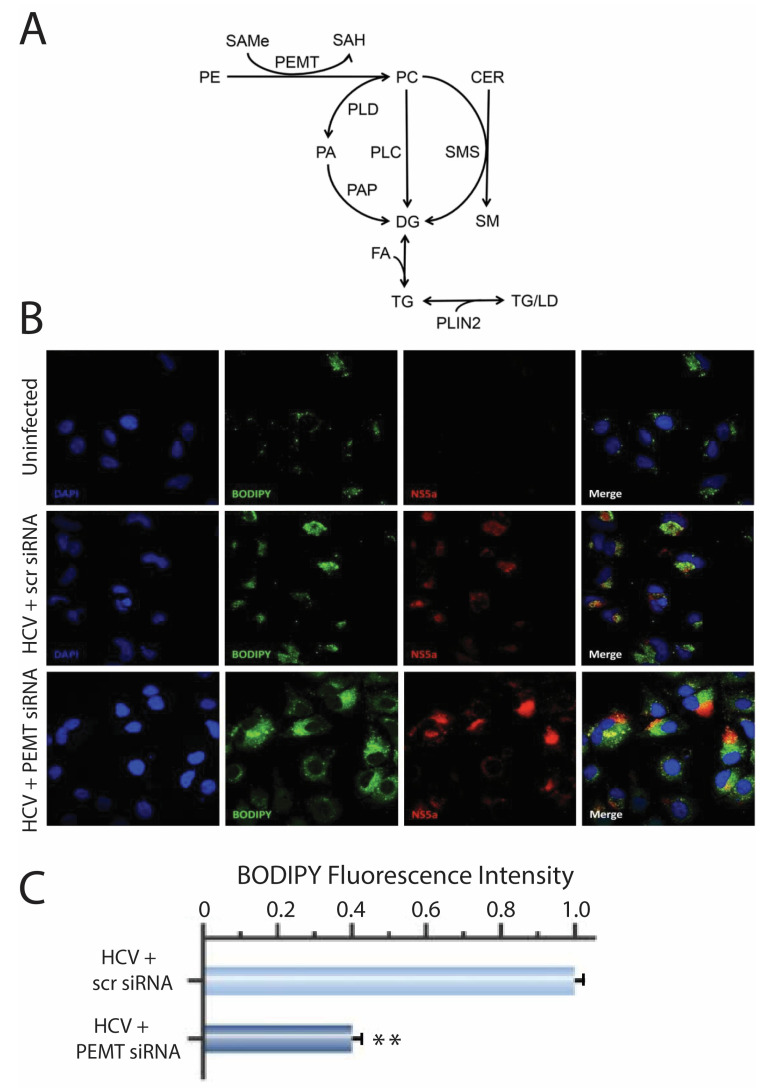
Involvement of PEMT in lipid synthesis. (**A**) Pathways involving PC and PEMT in lipid synthesis (reproduced with permission from [17]) (**B**) BODIPY staining (green) of neutral lipids in HCV-infected cells (NS5A positive, red) treated with 20 nM siRNA targeting PEMT or scrambled siRNA control. (**C**) Quantification of normalised fluorescent activity of BODIPY staining in HCV- infected cells treated with 20 nM siRNA targeting PEMT or scrambled RNA (** *p* < 0.01).

Phosphatidylcholine (PC) is an important mediator in the synthesis of triacylglycerol (TAG) (Figure 4A). We hypothesised that the mechanism by which PEMT regulates HCV replication is by controlling TAG synthesis. To test this hypothesis in the infected cells we examined the effects of knocking down PEMT on cellular lipids. It was not possible to generate sufficient siRNA-treated cells for HPTLC analysis, so we used a specific dye (BODIPY TM 493/503) to examine changes in the amount and distribution of the neutral lipid. The amount of intracellular lipid increased following HCV infection, but was significantly lower in the virus-infected cells treated with PEMT siRNA compared to the scramble controls (Figure 4B,C, *p* < 0.01). Consistent with the PEMT knock-down reducing HCV RNA (Figure 3G), we also observed reduced expression of HCV protein (NS5A) in cells treated with PEMT siRNA (Figure 4B).

### 2.5. PEMT Mediates HCV-Induced Expression of Lipid Genes and May Explain HCV Genotype Effects

**Figure 5:**
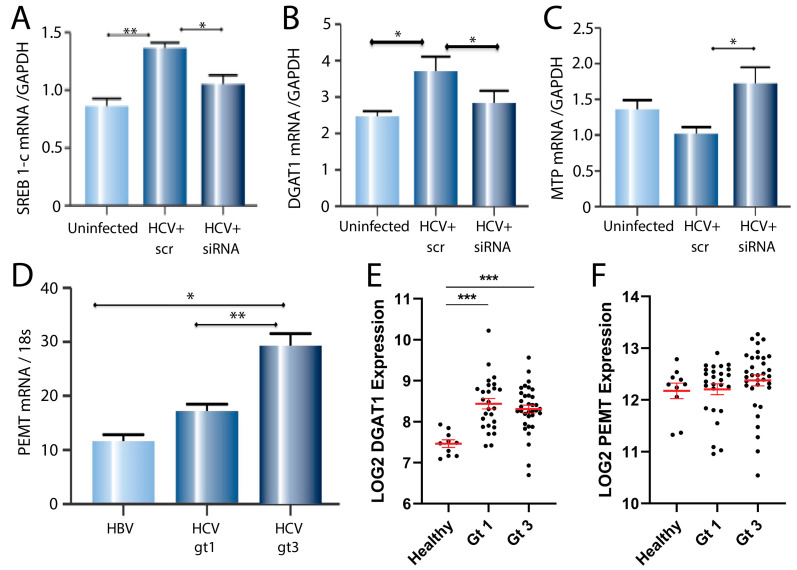
Expression of genes associated with lipid metabolism in response to HCV infection and PEMT knock-down. Real-time PCR quantification of (**A**) SREB 1-c, (**B**) MTP and (**C**) DGAT1 mRNA (normalised to GAPDH) in uninfected and HCV-infected cells treated with 20 nM siRNA targeting PEMT or scrambled siRNA control (* *p* < 0.05, ** *p* < 0.01). (**D**) Relative expression of PEMT mRNA (normalised to 18s) in liver biopsies from patients with HBV (*n* = 23), HCV-genotype 1 (*n* = 31) or genotype 3 (*n* = 24) infection (* *p* < 0.05, ** *p* < 0.01). (**E**) Relative expression of DGAT1 mRNA in liver biopsies from patients with healthy liver (*n* = 10), HCV-genotype 1 (*n* = 26) or genotype 3 (*n* = 33) infection (*** *p* < 0.001). Black circles represent individual data points. (**F**) Relative expression of PEMT mRNA in liver biopsies from patients with healthy liver (*n* = 10), HCV-genotype 1 (*n* = 26) or genotype 3 (*n* = 33) infection. Black circles represent individual data points.

A range of host factors involved in lipid metabolism are important for HCV replication, including serum response element binding protein (SREBP) 1-c, diacylglycerol acyltransferase 1 (DGAT1) and microsomal triglyceride transfer protein (MTP) [18,19,20]. To determine whether PEMT is involved in the virus-induced expression of these genes, we knocked down PEMT in HCV-infected cells using siRNA. Consistent with our hypothesis, the HCV-induced expression of SREBP 1-c (Figure 5A) and DGAT1 (Figure 5B) in infected cells was reversed by knocking down PEMT. There was a trend towards reduced MTP expression in the HCV-infected cells, with a significant increase following PEMT knock-down (Figure 5C).

Significantly different liver pathologies result from chronic infection with different HCV genotypes. Genotype 1 is commonly associated with insulin resistance [21] while genotype 3 is linked with higher rates of steatosis [22,23], but the mechanisms involved are not clear. To investigate if PEMT could be involved in the increased lipid accumulation with HCV genotype 3, we measured the PEMT expression in biopsy samples from patients with HCV-genotype 1 (*n* = 31) or genotype 3 (*n* = 24) infection. Biopsies from patients with hepatitis B virus (HBV) infection (*n* = 23) were included as extra controls, as HBV infection is not associated with insulin resistance or steatosis [24,25,26]. The expression of PEMT mRNA was similar in patients with HCV genotype 1 and HBV but was significantly higher in patients with HCV-genotype 3 infection compared to either HCV genotype 1 (*p* < 0.01) or HBV (*p* < 0.05) (Figure 5D).

To confirm our findings, we analysed the lipid gene expression in liver biopsies taken from patients with HCV-genotype 1 or genotype 3 infection using a publicly available dataset that we contributed to [27]. Consistent with our in vitro studies, there was an increased expression of DGAT1 in HCV-infected liver tissue compared to the healthy controls (Figure 5E). Consistent with our liver biopsy data (Figure 5D), there was a trend towards increased PEMT expression in liver tissue from patients with an HCV-genotype 3 infection, compared to genotype 1 or the healthy controls (Figure 5F).

## 3. Discussion

The major novel finding in our study is that PEMT plays a key role in HCV replication and its genotype-specific induction of steatosis, through the non-canonical synthesis of PC. We showed that knocking down PEMT inhibited HCV replication, reduced intracellular lipids and reversed the virus-induced induction of lipogenic genes. Importantly, knocking down PCYT1a, the rate limiting step in the choline-dependent PC synthesis pathway, did not reduce virus replication. This suggests that HCV stimulates PC synthesis via the non-canonical pathway involving PEMT. Finally, we demonstrated a higher PEMT expression in the livers of people infected with HCV genotype 3 than with genotype 1, providing a novel explanation for the genotype-specific differences in disease phenotype.

HCV modulates liver lipid metabolism to facilitate virus replication, but this can also cause hepatic steatosis, particularly in people with a genotype 3 infection [22,23]. The mechanisms involved are complex and only partially understood, but are key to understanding HCV pathogenesis. HCV promotes lipid synthesis in infected hepatocytes by stimulating SREBP 1-c [28], which promotes the synthesis of fatty acids and TAG by a range of enzymes including fatty acid synthase, stearoyl-CoA desaturase, acyl-CoA 6-desaturase, acetyl-CoA carboxylase and DGAT1 [18,19,20,29]. The intracellular accumulation of lipid is increased as the virus suppresses lipid oxidation by modulating nuclear peroxisome proliferator-activated receptor alpha and β-oxidation genes [30]. Finally, HCV reduces the secretion of lipids in VLDL by inhibiting MTP [31], leading to the accumulation of cellular lipid droplets [32].

Using precise HPTLC analysis of sub-cellular fractions, we demonstrated an increase in PC, PE, FC and DAG in the ER of HCV-infected cells, plus an increase in CE in whole cell lysates (lipid droplets), confirming previous semi-quantitative findings which used shotgun lipidomics [29]. The PC forms the membrane around lipid droplets [33], facilitates HCV RNA replication [34] and plays an essential role in the production of infectious HCV particles [35,36]. Most cell types synthesise PC using the choline-dependent pathway and previous studies have focused on this. HCV infection has been shown to alter choline uptake [37]. Human choline kinase-alpha, the first enzyme in the choline-dependent pathway, is an essential host factor for HCV replication [38,39]. However, this dependence is due to the direct interaction of human choline kinase-alpha with viral proteins in the replication complex, not to its effects on PC synthesis [40].

Uniquely, hepatocytes can synthesise PC directly from PE without choline using the alternative PEMT pathway [16]. We made the novel observation that the knock-down of PEMT in HCV-infected cells reduced viral RNA and protein, whereas inhibiting the choline-dependent pathway by knocking down its rate-limiting enzyme PCYT1 did not. This suggests that the HCV-stimulated increase in PC is not dependent on choline and involves the non-canonical PEMT pathway. Interestingly, PC accumulation has also been shown with several other positive-strand RNA viruses [41], so our findings may have implications beyond hepatitis C.

In addition to supporting virus replication, our results suggest that virus-induced PEMT could orchestrate hepatic steatosis in people with hepatitis C. We showed that knocking down PEMT in HCV-infected cells reduced the amount of lipid and reversed virus-induced changes in SREBP 1-c, DGAT1 and MTP, proposed mediators of steatosis in hepatitis C [18,19,20].

Finally, our results shed light on why the virus genotype affects steatosis. We have shown previously that HCV-genotype 3 infection is associated with increased rates of steatosis, independent of other metabolic risk factors [42]. The reason for this conundrum is unclear, although one study showed lower MTP activity in patients with genotype 3 infection [20]. Due to complex virus–host interactions, we did not attempt to model this in a cell culture but went straight to human tissue. We showed higher PEMT expression in liver biopsies from patients with HCV-genotype 3 infection than genotype 1, offering a plausible mechanism. This effect was virus-specific and consistent with the clinical phenotype as it was not observed in patients infected with HBV which, in contrast to HCV, does not cause steatosis [24,25,26]. Our observation that the virus-induced suppression of MTP expression can be reversed by knocking down PEMT further supports this hypothesis. Altogether our results suggest that a greater induction of PEMT in patients with genotype 3 infection promotes more hepatic steatosis, through the downstream effects on MTP, SREBP 1-c, DGAT1 and other key lipid genes.

**Figure 6:**
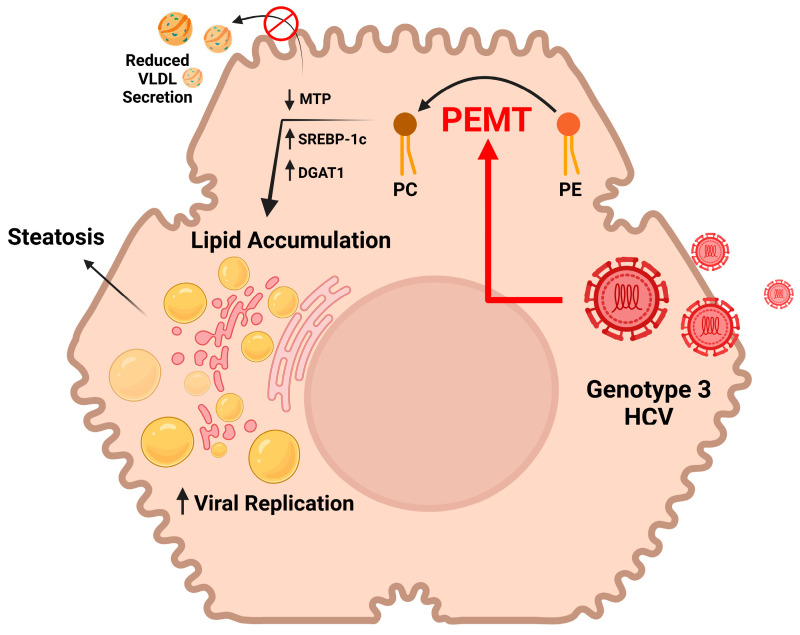
Proposed model for HCV-induced steatosis. HCV increases PC synthesis from PE by inducing PEMT. PC stimulates production of TAG by inducing SREBP-1c and DGAT1, while reducing secretion of VLDL by inhibiting MTP.

Thus, we propose a novel mechanism for HCV-induced steatosis through complementary mechanisms (Figure 6). (1) The increased expression of PEMT directly increases PC synthesis, stimulating the formation of cytoplasmic lipid droplets. (2) Increased cellular PC stimulates the production of TAG by increasing the expression of SREBP-1c and DGAT1. (3) Increased PEMT reduces the expression of MTP, inhibiting the secretion of VLDL and leading to the accumulation of fat in the liver.

In summary, our findings suggest that in chronic hepatitis C, PEMT plays a coordinating and critical role in both viral replication and the development of steatosis, by promoting the synthesis of PC. Targeting PEMT offers a novel approach to reduce steatosis and enhance antiviral therapy against hepatitis C and potentially other positive-strand RNA viruses.

## 4. Materials and Methods

### 4.1. Antibodies for Western Blotting and Immunocytochemistry

Rabbit polyclonal antibody against PEMT was obtained from LSBio (catalogue number LS-C80583). Sheep polyclonal antibody against NS5A was a kind gift from Prof. Mark Harris (University of Leeds, Leeds, UK).

### 4.2. Cell Culture and Production of HCV-Infected Cells (JFH1)

All cells used in this study were grown in Dulbecco’s modified eagle medium (DMEM) supplemented with 10% foetal calf serum (FCS) at 37 °C in 5% CO_2_. In vitro transcribed HCV RNA was generated from *Xba1*-linearised pJFH1 plasmid, a kind gift from Prof. Takaji Wakita (Tokyo Metropolitan Institute for Neuroscience, Tokyo, Japan), using the T7 RiboMAXTM Express RNAi System (Promega) [12]. Huh7 cells were transfected with viral RNA (10 μg) using electroporation at 0.34 kV, 974 μF. Electroporated cells were grown for at least two weeks and assayed at each passage for HCV infectivity by immunolabelling with anti-NS5A antibody. Only cultures which displayed > 90% infectivity were used for experiments.

### 4.3. Determining Virus Infectivity Using Tissue Culture Infective Dose (TCID50)

The TCID50 assay was performed as we have published it previously [43]. Briefly, supernatant from JFH1-infected Huh7 cells was added in 1:5 serial dilutions to uninfected Huh7 cells in 96-well plates. After 72 h, cells were fixed with methanol and stained for HCV with anti-NS5A antibody and secondary HRP-conjugated anti-sheep antibody. Infected cells were identified using the ImmPACT DAB Peroxidase Substrate (Vector laboratories) and examined with an Olympus CK2 inverted microscope at 20× magnification. A well was counted as positive if there was at least one infectious focus identified. The TCID50/mL was calculated as described in the Spearman and Kaerber method [44].

### 4.4. Endoplasmic Reticulum Enrichment

Endoplasmic reticulum enrichment was performed according to the protocol of Bozidis et al. (2007) [45]. Briefly, cells were harvested with trypsin, pelleted and resuspended in mannitol-tris-EDTA (MTE) buffer (250 mM mannitol, 10 mM Tris pH 7.4, 1 mM EDTA) containing protease inhibitors and phenylmethylsulfonyl fluoride (PMSF). Cells were lysed with tip sonication at 35% intensity (3 × 5 s pulses) and centrifuged at 14,000× *g* at 4 °C in a benchtop centrifuge. The supernatant was layered on top of a 1.3 M sucrose cushion and centrifuged at 35,000 rpm in a SW41 ultracentrifuge rotor for 1 h at 4 °C. A band containing ER was present at the interface with the sucrose layer which was extracted, diluted in MTE and pelleted at 35,000 rpm in an SW41 rotor for 1 h.

### 4.5. RNA Extraction and cDNA Synthesis

RNA was extracted using the Favorgen RNA extraction kit and cDNA was generated from 500 ng RNA using MMLV reverse transcriptase (Promega), according to the manufacturers’ protocols. All real-time PCR reactions were carried out on a Corbett Rotorgene 6000. PEMT (forward primer 5′-GGATGAAGAGATCTGGGAACC-3′, reverse 5′-ATGACGGGCAGCCACAAAG-3′), PYCT1a (forward 5′-TCCCGAATTCATTGGAAG-3′, reverse 5′-TGAAGCGACAGGTTTCTTCTG-3′), ISG15 (forward 5′-CGCAGATCACCCAGAAGATC-3′, reverse 5′-GCCCTTGTTATTCCTCACCA-3′), OAS3 (forward 5′-GTCAAACCCAAGCCACAAGT-3′, reverse 5′-GGGCGAATGTTCACAAAGTT-3′), SREB 1-c (forward 5′-GCCATGGATTGCACTTT-3′, reverse 5′-CAAGAGAGAGCTCAATG-3′) and GAPDH (forward 5′-CCATTCAATGACCCTTGTTG-3′, reverse 5′-CTGGGATTTCCATTGATGACAAG-3′) were quantified using SYBR green. HCV 5′-UTR (Pa03453408_s1) and MTP (Hs00165177_m1) were quantitated using primer probe sets obtained from Applied Biosystems. All real-time PCR calculations were based on triplicates of three separate biological samples.

### 4.6. Lipid Extraction

Cellular lipids were extracted using a modified Bligh–Dyer protocol [46]. Briefly, cell/organelle pellets were suspended in a volume of phosphate-buffered saline (PBS) approximately five times the pellet volume. Methanol and chloroform were added to give a methanol:choloroform:saline ratio of 2:1:0.8. The mixture was then homogenised and an equal volume of 1 M NaCl:50 mM HCl was added, followed by an equal volume of chloroform to induce phase separation. The mixture was then centrifuged at 800× *g* in a swing bucket rotor for 10 min. The lower (chloroform) phase was collected and dried under a stream of nitrogen and stored in an airtight vial at −80 °C.

### 4.7. High-Performance Thin-Layer Chromatography (HPTLC)

Plates for HPTLC were pre-washed with methanol/ethylacetate (6:4, *v*/*v*) and activated at 110 °C for at least 30 min before loading. Extracted lipids were dissolved in chloroform/methanol (2:1, *v*/*v*) and then loaded onto HPTLC plates with a series of lipid standards under nitrogen using the Automatic TLC sampler 4 (ATS4) and developed in the CAMAG AMD2 (CAMAG, Wilmington, NC, USA) [47]. For neutral lipids, HPTLC plates were developed twice with dichloromethane/ethyl acetate/acetone (80:16:4, *v*/*v*/*v*) to 40 and 55 mm, then sequentially three times with hexane/ethyl acetate to 68mm (90:10, *v*/*v*), 80 mm (95:5) and 90 mm. Phospholipids were developed according to Weerheim et al. (2002) [48] with modifications [11] using a two-step separation. HPTLC plates were first developed with dichloromethane/ethyl acetate/acetone (80:16:4, *v*/*v*/*v*) to 90 mm, dried under a vacuum for 6 min, then developed again to 90 mm with chloroform/ethyl acetate/acetone/isopropanol/ethanol/methanol/water/acetic acid (30:6:6:6:16:28:6:2, by volume).

### 4.8. Chromatogram Staining and Visualisation of HPTLC Plates

After development, plates were sprayed uniformly with 10% cupric sulphate in 8% aqueous phosphoric acid [11,49], allowed to dry for 10 min at room temperature and then heated to 145 °C for 10 min. Imaging of cupric-sulphate charred plates was carried out using the Luminescent Image Analyser LAS-4000 (Fuji Film, Tokyo, Japan). All images were analysed using Multi Gauge V3.0 software.

### 4.9. Lipid Mass Spectrometry

Bands of interest were scraped off HPTLC plates, solubilised and centrifuged to remove TLC particles, then transferred into glass LC-MS vials. Samples were analysed using both positive and negative electrospray ionisation on a Thermo Fisher Scientific (Waltham, MA, USA) QEXactive Plus mass spectrometer. The sample was injected using an ULTIMATE 4000 system (Dionex) and chromatographed on a Waters CSH 2.1 × 100 mm C18 column, according to the method described by Castro-Perez et al. (2011) [50]. The instrument was run at a 140,000-resolution full scan, followed by six lower-resolution DDA scans on the six most intense peaks identified in the full scan. Data were interrogated using Lipid Search software (Thermo Fisher Scientific). Samples were analysed multiple times to allow statistically significant comparisons to be made.

### 4.10. Liver Biopsy Gene Expression Analysis

PEMT expression was measured in liver biopsies from patients in our clinics using real-time qPCR. Liver biopsy samples were collected from patients with chronic viral hepatitis, due to either HBV (*n* = 23), HCV genotype 1 (*n* = 31) or genotype 3 (*n* = 24) [51]. Ethics approval was obtained from the Human Research Ethics Committees of Sydney West Area Health Service (Sydney, Australia) and the University of Sydney (Sydney, Australia). All subjects provided written informed consent (HREC2002/12/4.9(1564)) prior to the research.

An independent validation cohort dataset E-MTAB-7751 [27] was downloaded from Array Express: https://protect-au.mimecast.com/s/fYlmC2xMQzipDRJ9wSBPFJy?domain=ebi.ac.uk/ (accessed on 14 March 2022).

### 4.11. Statistical Analysis

For in vitro studies and single gene expression analysis, Student’s t-test was used to compare groups, with statistical significance defined by *p* < 0.05. Data are summarised as column graphs or individual data points, with error bars showing the standard error of the mean (SEM).

For gene expression array analysis, public array data analysis was performed in the R statistical environment (V4.1, R Core Team (2021). R: A language and environment for statistical computing. R Foundation for Statistical Computing, Vienna, Austria. URL https://www.R-project.org/ (accessed on 14 March 2022)). Gene expression was compared between sample groups by anova in tidyverse (Alboukadel Kassambara (2020). ggpubr: ‘ggplot2’ Based Publication Ready Plots. R package version 0.4.0. https://CRAN.R-project.org/package=ggpubr (accessed on 14 March 2022)) [52].

## Data Availability

The data presented in this study are available on request from the corresponding author. The data are not publicly available due to restrictions from our Human Research Ethics Committees.

## Supplementary Materials

The supporting information can be downloaded at https://www.mdpi.com/article/10.3390/ijms24108781/s1.
