# Supplementary material for: PEMT Mediates Hepatitis C Virus-Induced Steatosis, Explains Genotype-Specific Phenotypes and Supports Virus Replication
Source: Int J Mol Sci. 2023 May 15;24(10):8781. doi: 10.3390/ijms24108781 (PMC10218061; doi:10.3390/ijms24108781)
Supplement: Supplementary file 1 [file ijms-24-08781-s001.zip › ijms-2373751-supplementary.pdf]

Supplementary Figures

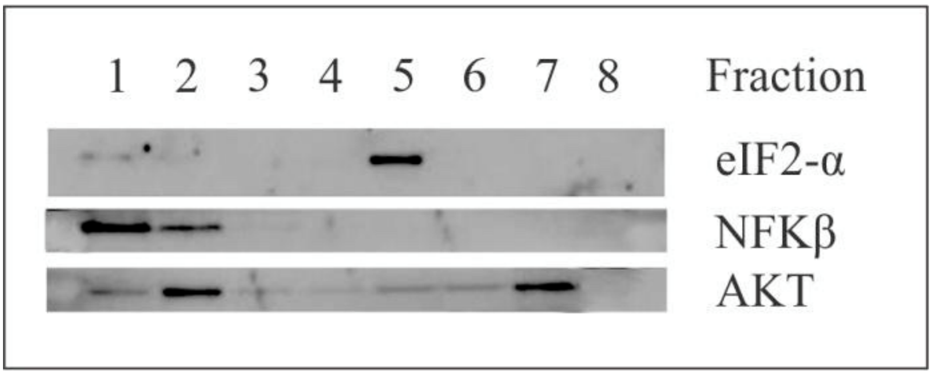

Figure S1: Western blot analysis of subcellular proteins to verify the purity of isolated ER. Subcellular fractionation of JHF1 infected and control Huh-7 cells was performed by discontinuous sucrose gradient centrifugation. Protein (20 μg) from each fraction was separated by 10% SDS PAGE and transferred onto PVDF membranes. Blotted proteins were probed for markers of ER (eIF2-α), nucleus (NF-κB) and cytosol (AKT).
